# Asproinocybaceae fam. nov. (Agaricales, Agaricomycetes) for Accommodating the Genera *Asproinocybe* and *Tricholosporum,* and Description of *Asproinocybe sinensis* and *Tricholosporum guangxiense* sp. nov.

**DOI:** 10.3390/jof7121086

**Published:** 2021-12-17

**Authors:** Guang-Fu Mou, Tolgor Bau

**Affiliations:** Engineering Research Centre of Chinese Ministry of Education for Edible and Medicinal Fungi, Jilin Agricultural University, Changchun 130118, China; guangfuMOU@163.com

**Keywords:** new taxa, phylogeny, taxonomy, karst areas, Tricholomataceae

## Abstract

*Asproinocybe* and *Tricholosporum* are not well known, and their placement at the family level remains undetermined. In this study, we conducted molecular phylogenetic analyses based on nuc rDNA internal transcribed spacer region (ITS) and nuc 28S rDNA (nrLSU), and a dataset with six molecular markers (ITS, LSU, RNA polymerase II largest subunit (RPB1), RNA polymerase II second largest subunit (RPB2), 18S nuclear small subunit ribosomal DNA (nrSSU), and translation elongation factor 1-alpha (TEF1-α)) using Bayesian (BA) and Maximum Likelihood (ML) methods, we found that the species of *Asproinocybe* and *Tricholosporum* formed an independent family-level clade (0.98/72). Asproinocybaceae fam. nov., a new family, is established here for accommodating this clade. Two new species, *Asproinocybe sinensis* and *Tricholosporum guangxiense*, from subtropical and tropical karst areas of China, are also described here.

## 1. Introduction

The genera *Asproinocybe* R. Heim (1970) and *Tricholosporum* Guzmán (1975) are usually placed in Tricholomataceae due to their tricholomatoid basidioma [1,2,3,4,5,6].

*Asproinocybe* was originally described as *Leucinocybe* Heim (1969) and typified by *Leucinocybe lactifera* Heim (1969) [7]. *Leucinocybe* is mainly characterized by indigo or violet basidiomata, hyaline and tuberculous spores, and the presence of laticifers [7]. However, *Leucinocybe* was used by Singer for accommodating *Mycena lenta* Maire, meaning that *Leucinocybe* Heim (1969) is invalid. Later, Heim (1970) proposed the new name *Asproinocybe*, typified by *Asproinocybe lactifera* [8]. In the current sense, the genus is characterized by tricholomatoid; distinctive purplish, violaceous, or lilac-vinaceous basidioma; lamellae bruising reddish when damaged; spore hyaline and with irregularly tubercle; with laticifers present [6,7,8,9].

*Tricholosporum* was erected based on the combination of *Tricholoma goniospermum* Bres. (as type) and *Tricholoma porphyrophyllum* S. Imai [both from *Tricholoma* section *Iorigida* Singer (1945)] and the description of *Tricholosporum subporphyrophyllum* Guzmán, due to its cruciform spores and lamella with lilac or purple pigments [1,10].

The segregation of the two genera was latter recognized, but the combinations were considered invalid because the original publications of the basionyms were not provided [11]. Then, *Tricholosporum goniospermum* and *T. porphyrophyllum*, as well as *Tricholosporum atroviolaceum* and *Tricholosporum pseudosordidum* were added to *Tricholosporum* [11].

The independence of *Asproinocybe* and *Tricholosporum* was long debated. Singer recognized *Asproinocybe* considered in tribe Tricholomateae [12], but *Tricholosporum* was considered synonym of *Tricholoma* [12,13,14,15,16]. On the other hand, *Asproinocybe* and *Tricholosporum* were also considered as independent entities [2]. This opinion has been widely recognized [3,4,6].

By now, eight species recognized in *Asproinocybe*. Vicente et al. constructed a key for the species [6,7,8,9,17]. With 14 species recognized in *Tricholosporum*, Vicente et al. and Angelini et al. published a key for the species [4,5,9].

Regarding the placement at the family level, *Asproinocybe* was not indicated as belonging to a specific family when it was established: it was only compared with *Lyophyllum* [7,8]. In 1977, Heinemann assigned *Asproinocybe russuloides* to Tricholomataceae, later followed by Guzmán and Lebel et al. [3,6,18]. *Tricholosporum* was established in Tricholomataceae [1]. Thus, *Asproinocybe* and *Tricholosporum* were placed in Tricholomataceae for a long time based on morphological consideration.

However, more recently, phylogenetic studies are increasingly showing that they should not be placed in Tricholomataceae [6,19,20]. The first phylogenetic approach recovered *Tricholosporum* within Entolomataceae based on ITS dataset and within Tricholomataceae (s.l.) based on LSU [19].

Later, Angelini et al. conducted a more comprehensive phylogenetic study on the relationships between *Tricholosporum* and Tricholomatineae [20]. They used ITS, LSU, SSU, and RPB2 DNA sequences to evaluate the phylogenetic position of *Tricholosporum* within the clade of tricholomatoid fungi. Their analysis showed a weak relationship of *Tricholosporum* in the clade of Tricholomataceae, and an isolated position of this genus within the Tricholomatineae. Their tree, based on SSU and RPB2 sequences, placed *Tricholosporum* in the Entolomataceae/Lyophyllaceae, whereas the LSU and ITS trees placed *Tricholosporum* within a group of morphologically heterogeneous species such as *Macrocybe gigantea*, *Clitocybe fellea*, *Pleurocollybia brunnescens*, and *Callistosporium* spp. The tree, combined RPB2-SSU-LSU sequences, showing a relationship of *Tricholosporum* with the clade Entolomataceae, Lyophyllaceae, the Clytocybe/Lepista/Collybia, and the callistosporoid groups, but the relationship was poorly resolved and had weak bootstrap support [20].

Both studies conducted a phylogenetic analysis of *Tricholosporum* to find a suitable placement at the family level but failed. They confirmed that *Tricholosporum* should not be placed in Tricholomataceae. However, the researchers only used a single or a few species of *Tricholosporum* in the phylogenetic analysis. Heaton and Kropp postulated that using RPB1 would probably lead to a better understanding of the phylogenetic placement of *Tricholosporum* [19].

Only in 2020 were new species from *Asproinocybe* found and included in phylogenetic analysis [6]. Lebel et al. found two new species from *Asproinocybe* and conducted a phylogenetic analysis based on ITS sequences only. They found that the species from *Asproinocybe* and *Tricholosporum* formed a clade, which suggested weak support for Biannulariaceae but strong support for a restricted Catathelasmataceae and for a clade with *Infundibulicybe*, *Anupama*, *Guyanagarika*, Tricholomataceae sp., *Asproinocybe*, and *Tricholosporum* as sisters to Catathelasmataceae [6]. In a restricted multimarker analysis of a broad selection of taxa from Lyophyllaceae, Entolomataceae, and Tricholomatoid agarics, support for the placement of *Asproinocybe* and *Tricholosporum* in a broad Tricholomataceae was weak [6].

Lebel et al. demonstrated the phylogenetic relationship between *Asproinocybe* and *Tricholosporum* for the first time but could not solve the phylogenetic problems at the family level, and confirmed that the idea of *Tricholosporum* being distinct from *Asproinocybe* was problematic. All the abovementioned phylogenetic studies were either conducted using only a single marker or included only a single species, which prevented the determination of the relationship between *Asproinocybe* and *Tricholosporum* and of the placement at the family level. A more comprehensive sampling of *Asproinocybe* and *Tricholosporum* and involving multimarker data in the phylogenetic analysis may help to solve these problems.

The aim of this study is to determine the family-level placement of *Asproinocybe* and *Tricholosporum* and to further discuss the relationships between *Asproinocybe* and *Tricholosporum* from morphology and phylogeny perspectives. Two new species from *Asproinocybe* and *Tricholosporum* are described.

## 2. Materials and Methods

### 2.1. Sampling, Morphological Observations, and Descriptions

Specimens were collected from the Yachang Orchidaceae National Nature Reserve, Leye County, Baise city, Guangxi Province, China (24°44′16″–24°53′58″ N, 106°11′31″–106°27′04″ E), at an elevation of about 1050 m, and the Nongang National Nature Reserve, Ningming County, Chongzuo City, Guangxi Province, China (22°13′56″–22°33′09″ N, 106°42′28″–107°04′54″ E), at an elevation of about 200 m. One specimen was collected from Changchun City, Jilin Province, China. The specimens were dried in silica gel or an oven at 50 °C. The dried specimens were preserved in the Herbarium of Mycology of Jilin Agricultural University (HMJAU) and Herbarium of Guangxi Institute of Botany (IBK) (see Supplementary Materials Table S1, in bold). The macroscopic characteristics were based on the fresh specimens. Color codes were assigned according to Kornerup and Wanscher [21]. Microscopic characteristics were obtained from dried specimens that were examined using a light microscope (Olympus BX53, Olympus, Tokyo, Japan). Color microscopic photos were taken with an Olympus camera (Olympus EP50, Olympus, Guangzhou, China). SEM photos were taken by scanning electron microscopy (ZEISS EV018, ZEISS, Cambridge, UK). Measurements were performed on the tissues mounted in pure water or 5% KOH solution. The tissues were stained with 1% Congo Red solution or Lactate Carbolic Cotton Blue. Amyloid reactions were tested in Melzer’s reagent. For the descriptions of microscopical features, we referenced Jian et al., namely, the term [n/m/p], which indicates n basidiospores from m basidiomata of p collections. The dimensions for the basidiospores were given using notation of the form (a–) b–c (–d); the range b–c contains a minimum of 90% of the measured values; extreme values, i.e., a and d, are given in parentheses; Q denotes the length/width ratio of a basidiospore from the side view; Q_avg_ is the average Q of all the specimens ± the sample standard deviation [22].

### 2.2. DNA Extraction, PCR, and Sequence Amplification

Total genomic DNA was extracted from the dried specimens using a NuClean Plant Genomic DNA kit (ComWin Biotech, CW0531M, Taizhou, China), following the manufacturer’s instructions. The primer pairs ITS1/ITS4 or ITS4/ITS5 [23], LR0R/LR7 or LR0R/LR5 [24], gRPB1-A/fRPB1-C rev [25], fRPB2-5F/fRPB2-7Cr [26], PNS1/NS41 [27], and EF1-983F/EF1-1567R [28] were used to amplify the ITS, nrLSU, RPB1, RPB2, nrSSU, and TEF1-α sequences, respectively. Polymerase chain reaction (PCR) was performed on a Bio-Rad T100^TM^ Thermal cycler (Bio-RAD Inc., Hercules, CA, USA). The amplification reactions were performed in a 30 µL reaction mixture using the following final concentrations or total amounts: 2 µL of template DNA, 15 µL of 2× Es Taq MasterMix (Dye, ComWin Biotech, CW0690H, Taizhou, China), 1.5 µL of each primer, and 10 µL of ddH_2_O (double-distilled water).

The PCR procedure was performed under the following conditions: 95 °C for 4 min and then 35 cycles of denaturation at 94 °C for 60 s, annealing at 53 °C (ITS, nrLSU)/55 °C (nrSSU, TEF1-α) for 60 s, 2 min at 55 °C, an increase of 1 °C/5 s to 72 °C (RPB1, RPB2), and extension at 72 °C for 90 s, with a final extension at 72 °C for 10 min. The PCR products were electrophoresed on a 1% agarose gel with known standard DNA markers. The DNA sequencing was performed by Shenggo Biological Technology Co. Ltd. (PE Applied Biosystems, ABI 3730XL, Foster, CA, USA). The chromatograms were checked in bioEdit v7.2.5 [29] to ensure that every single base was of good quality, and we conducted a BLAST search using the National Center of Biotechnology Information (NCBI) database to confirm that the sequencing results matched the specimens and then submitted the sequences to GenBank (for the GenBank accession numbers, see Supplementary Materials Table S1 in bold).

### 2.3. Data Analysis

In this study, we used the sequences of 119 specimens from 6 families, 41 genera, and 86 species, of which 33 sequences of seven specimens belonged to the new taxon and three specimens of 10 sequences were new. The sequences downloaded from GenBank were mainly from Matheny et al., Co-David et al., Hofstetter et al., Sánchez-García et al., Alvarado et al., Raj et al., Vizzini et al., Lebel et al. and Jian et al. [6,22,30,31,32,33,34,35,36,37,38] (those in bold in Supplementary Materials Table S1 were newly sequenced). A six-marker (ITS, nrLSU, RPB1, RPB2, nrSSU, and TEF1-α) dataset was used for molecular phylogenetic analyses to confirm the phylogenetic placement of the genera *Asproinocybe* and *Tricholosporum* at the family level. We used a total of 46 sequences (see Table S1 for the GenBank Accession numbers marked with asterisks) of 27 specimens from *Asproinocyve*/*Tricholosporum* and related species’ ITS and nrLSU sequences for molecular phylogenetic analyses to confirm the new taxon’s phylogenetic placement within the genera.

The sequences of the six markers were aligned separately with online MAFFT using the default settings [39]. Prior to phylogenetic analysis, ambiguous sequences at the start and the end were deleted and gaps were manually adjusted to optimize the alignment using the default parameters in BioEdit v7.2.5 [29]. Multimarkers were concatenated as a combined file using SequenceMatrix [40]. Sequences of *Suillus pictus*, *Pseudoarmillariella ectypoides*, and *Ampulloclitocybe clavipes* were used as the outgroup for the six-marker (partial ITS, nrLSU, RPB1, RPB2, nrSSU, and TEF1-α) dataset, for which we referred to Vizzini et al. [36]. Sequences of *Callistosporium luteoolivaceum*, *Callistosporium xanthophyllum*, *Lepista irina*, and *Lepista nuda* were used as the outgroup for the partial ITS + nrLSU dataset because of their close relationship and similar morphology [6,20]. The final concatenated sequence alignments were deposited in TreeBase https://treebase.org/treebase-web/home.html (accessed on 28 October 2021) with the submission ID 28935 for the six markers and submission ID 28967 for the partial ITS + nrLSU dataset.

MrModeltest v.2.3 was used to estimate the optimal model [41]. The best-fit model used for Bayesian inference (BI) analysis for the combined six-marker data subset (the six-marker dataset was treated individually), was the same, was the GTR + I + G model; for the combined two-marker data subset, the ITS subset (1–708 bp), was the GTR + G model; for the nrLSU subset (709–1589 bp), we used the GTR + I + G model. Maximum likelihood (ML) bootstrap analysis was performed under the GTRGAMMA model (the six-marker dataset and the two-marker dataset were treated as a whole).

For the dataset in Supplementary Materials (Figure S2), we used the same processing as for the above two-marker dataset. For the dataset in Supplementary Materials (Figure S1), the same best-fit model used for BI analysis was the same for both the ITS subset and nrLSU subset: the GTR + G model; the processing of the others was the same as that for the above two-marker dataset.

Bayesian inference analysis was performed with MrBayes v.3.2.6; with 0.2 million generations (partial ITS + nrLSU) and for 15 million generations (partial ITS + nrLSU + RPB1 + RPB2 + nrSSU + TEF1-α), with four chains and sampling every 100th generation four Markov chains (MCMC) were run, until the split deviation frequency value was <0.01 [42]. Maximum likelihood (ML) bootstrap analysis was performed with a rapid bootstrapping algorithm and 1000 replicates, followed by an ML tree search in raxmlGUI 2.0 [43]. The tree was visualized using Figtree v1.4.3 and edited by means of Adobe Photoshop CS6 [44]. Branches that received bootstrap support for Maximum Likelihood (BS) and Bayesian posterior probabilities (BPP) greater than or equal to 70% (BS) and 0.95 (BPP) were considered as significantly supported.

## 3. Results

### Phylogenetic Analyses

**Figure 1:**
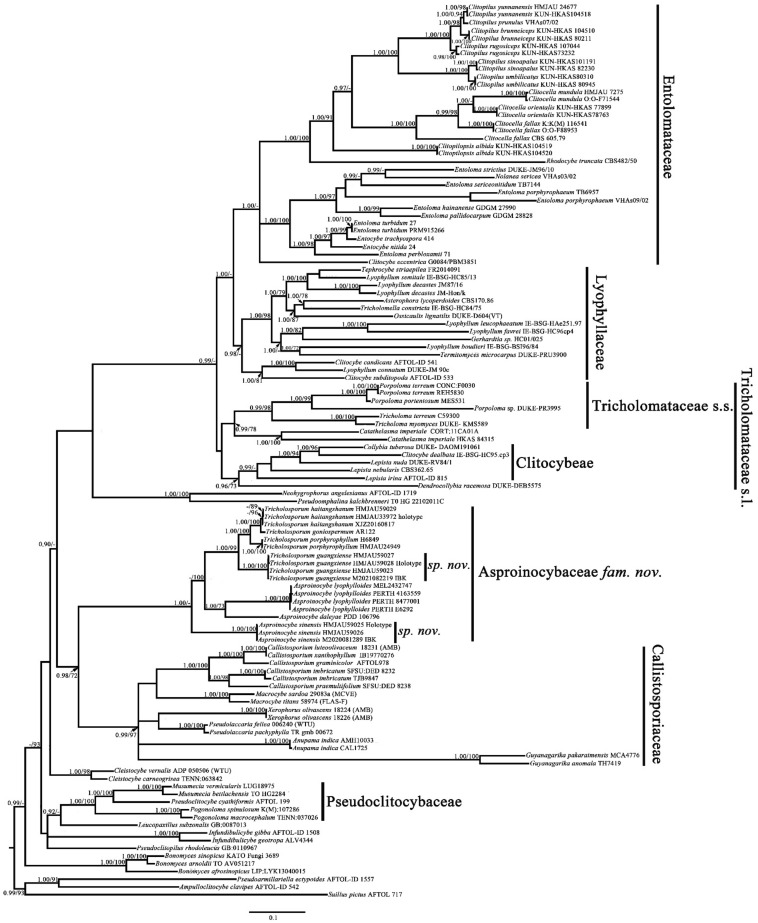
Phylogenetic tree inferred from partial ITS + nrLSU + RPB1 + RPB2 + nrSSU + TEF1-α sequences, showing phylogenetic relationships of Asproinocybaceae and related taxa (with *Suillus pictus*, *Pseudoarmillariella ectypoides*, and *Ampulloclitocybe clavipes* as outgroups). Bayesian inference (BPP ≥ 0.90) and maximum likelihood support values (ML ≥ 70) are shown (BPP/ML).

**Figure 2:**
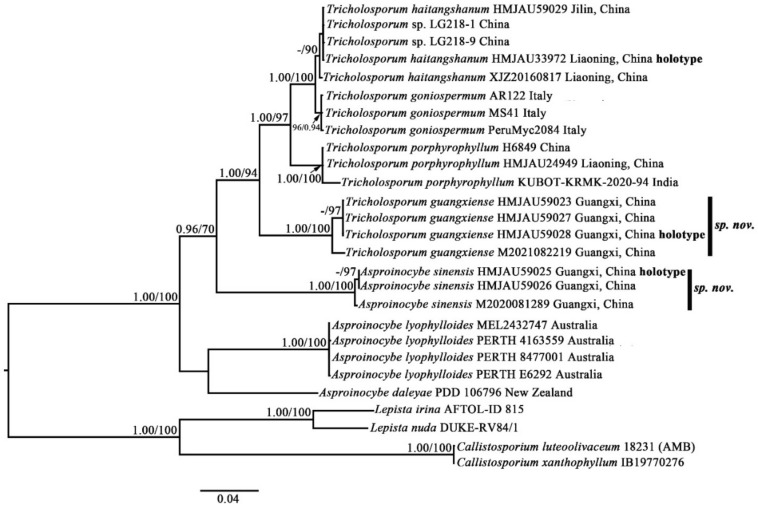
Phylogenetic tree inferred from partial ITS + LSU sequences showing phylogenetic relationships of *Asproinocybe sinensis* and *Tricholosporum guangxiense* within genus. Bayesian inference (BPP ≥ 0.90) and maximum likelihood support values (ML ≥ 70) are shown (BPP/ML).

The six-marker dataset combining partial ITS (1–771 bp) + nrLSU (772–1746 bp) + RPB1 (1747–3116 bp) + RPB2 (3117–4164 bp) + nrSSU (4165–4888 bp) + TEF1-α (4889–5477 bp) had an aligned length of 5477 total characters including gaps. The partial ITS + nrLSU dataset had an aligned length of 1589 (ITS subset: 1–708 bp; nrLSU subset: 709–1589 bp) total characters including gaps. For the six-marker and the partial ITS + nrLSU datasets, BI analysis generated a topology similar to that of ML analysis. The best trees obtained from the BI and ML analyses with bootstrap values for BPP and BS are shown in Figure 1 and Figure 2 (topology of Bayesian tree).

The topology of the six-marker dataset grouped into seven main clades: Entolomataceae (1.00/-), Lyophyllaceae (0.98/-), Tricholomataceae s.s. (0.99/98), Clitocybeae (0.99/-), the clade formed by *Tricholosporum* and *Asproinocybe* (1.00/-), Callistosporiaceae (0.99/97), and Pseudoclitocybaceae (1.00/100). Both the BI and ML analyses provided significant support (0.98/72) for a monophyletic origin of the *Tricholosporum* and *Asproinocybe* clades and the family Callistosporiaceae. Given these results, a new family name is proposed to accommodate the *Tricholosporum* and *Asproinocybe* clades.

Within the *Tricholosporum* and *Asproinocybe* clades, our specimens form two distinct clades, and both clades received significant support (1.00/100), indicating that they represent two new species.

The topology in Figure 2 does not form two clades of independent genera. However, when we removed the sequences from *Asproinocybe sinensis* or the sequences from *A. lyophylloides* and *A. daleyae* and used the rest of the dataset in Table S1 for the GenBank Accession numbers marked with asterisks to reconstruct the phylogenetic tree, the species of *Tricholosporum* and *Asproinocybe* form two independent clades. These results are shown in Figures S1 and S2 (Supplementary Materials).

The partial ITS + nrLSU phylogeny results for the *Tricholosporum* and *Asproinocybe* clades are similar to those of the six-marker dataset, which show that our specimens form two independent lineages and received strong statistical support. In Figure 2, different specimens of *Asproinocybe sinensis* have 1.00/100 or-/97 (BPP/BS) statistical support, and this clade of species has 0.96/70 (BPP/BS) statistical support. *Tricholosporum guangxiense* received 1.00/100 or-/97 (BPP/BS) and 1.00/94 statistical support. The results in Figures S1 and S2 (Supplementary Materials) are similar to those in Figure 2.

## 4. Taxonomy

***Asproinocybaceae*** T.Bau et G.F.Mou, **fam. nov.**

Mycobank No: MB841852

Etymology: From the type genus *Asproinocybe*.

Description: Habit tricholomatoid. *Basidiomata* with distinctive purplish, violaceous, or lilac-vinaceous colors. *Pileus* broadly convex, subumbonate to flat-hemispherical, becoming plane to depressed with age, margin smooth or with light and short stripes, entire, incurved at first then straight, surface at first fibrillose-felted (due to very thin, white hairs) then finely velvety but smooth toward the center, nonviscid, or subviscidus; with varying degrees of purplish, violaceous, or lilac-vinaceous colors in surface, especially near the margin, center more or less yellowish, yellowish ochre, yellowish brown, brown to dark brown colors. Context firm, white or whitish, becoming greyish or cream yellowing. *Lamellae* adnate, adnexed, sinuate or emarginate to free, sometimes with small decurrent tooth; *lamellulae* exist; margins smooth or unevenly serrate; close to crowded or crowded; pale violet to deep violet or greyish violet, bruising reddish or pale brown when damaged. *Stipe* solid to fistulose-hollow, cylindric to slightly clavate, central, pale violet, violet, greyish violet to bluish violaceous when fresh, covered by white to pale violet flocculose pruina, bruising dull, fading to whitish with age. *Base* usually with white rhizomorphs. *Odor* not distinct or fragrant. *Taste* not distinct or bitter or sour. *Spore-print* white.

*Basidiospores* hyaline, colorless, inamyloid, thin-walled, cyanophilous or not, subglobose to subellipsoid, tuberculate to stellate (*Asproinocybe*), or cruciform to stauriform (*Tricholosporum*), usually with a single large oil-drop. *Basidia* cylindric to narrowly clavate, two sterigmate or four sterigmate, thin-walled, colorless. *Cheilocystidia* and *pleurocystidia* usually similar, present or absent, oblong to ellipsoid, utriform, ampullaceous, fusiform or clavate, with a swollen base and a neck, acute or mucronate at apex, thin-walled or occasionally thick-walled, colorless or golden brown, or sometimes with pinkish violet content or grey-violet pigment. *Hymenophoral trama* regular, inamyloid, not dextrinoid, thin-walled. *Pileipellis* consisting of a cutis of loosely interwoven, cylindric to clavate hyphae, smooth or with incrustation. *Clamp connections* present or absent. *Laticifers* present, both in *Asproinocybe* and *Tricholosporum*.

Type genus: *Asproinocybe* R. Heim, Revue Mycol., Paris 34(4): 343 (1970).

Habit: Scattered or gregarious on broad-leaved forests soil, usually found in summer or autumn.

Genera included: *Asproinocybe* R. Heim, *Tricholosporum* Guzmán

Distribution: *Asproinocybe* mainly distributed in tropics, whereas *Tricholosporum* is widespread [45].

Notes: Our phylogenetic analysis results (based on partial ITS + nrLSU + RPB1 + RPB2 + nrSSU + TEF1-α) show that *Asproinocybe* and *Tricholosporum* form a single family-level clade and received strong statistical support (BPP = 0.98, BS = 72), and the clade is a sister to the Callistosporiaceae clade, which is in agreement with previously published phylogenetic results [6,20]. Taking all of the phylogenetic and morphological results into account, a new family, *Asproinocybaceae* fam. nov., is proposed for the *Asproinocybe*/*Tricholosporum* clade.

The species of *Asproinocybe* and *Tricholosporum* are very similar in appearance: they can only be differentiated by the shape of the basidiospores. Some mycologists have discussed the split [2,6]. Our phylogenetic results (Figure 1 and Figure 2) show that the species of *Asproinocybe* and *Tricholosporum* always group together, but they do not form two single clades. However, when we removed the sequences from *Asproinocybe daleyae* and *Asproinocybe lyophylloides* and used the rest of the dataset in Table S1 (in Supplementary Materials, the GenBank Accession numbers marked with asterisks) to reconstruct the phylogenetic tree, the species of *Asproinocybe* and *Tricholosporum* clearly formed two single clades (Figure S1). However, when we removed the sequences of *Asproinocybe sinensis* and used the rest of the dataset in Table S1 (GenBank Accession numbers marked with asterisks) to reconstruct the phylogenetic tree, the species of *Asproinocybe* or *Tricholosporum* clearly formed two single clades (Figure S2).

The morphological characteristics of our specimens (*Asproinocybe sinensis*) meet the definition of *Asproinocybe*; therefore, they must belong to *Asproinocybe*. Regarding why it did not form a single clade with *Asproinocybe daleyae* and *Asproinocybe lyophylloides*, we postulate that this may be due to the lack of sampling of species from *Asproinocybe*. When more species from *Asproinocybe* are included in the phylogenetic analysis, these questions may be able to be answered.

Thus, taking the results of Figures S3 and S4 and the stable shape of spores into account, we still treat *Tricholosporum* as being distinct from *Asproinocybe*.

***Asproinocybe*** R. Heim, Revue Mycol., Paris 34(4): 343 (1970).

Basionym: *Leucinocybe* Heim, *Cah. de La maboké*, VII, 2, 1969, p. 83.

Etymology: From the tuberculate basidiospores, similar to *Inocybe* but colorless.

Type species: *Asproinocybe lactifera* R. Heim 1970.

Basionym: *Leucinocybe lactifera* Heim, *Cah. de La maboké*, VII, 2, 1969, p. 83–85.

Ecology and distribution: Scattered or gregarious in broad-leaved forest soil, mainly distributed in the tropics.

**Figure 3:**
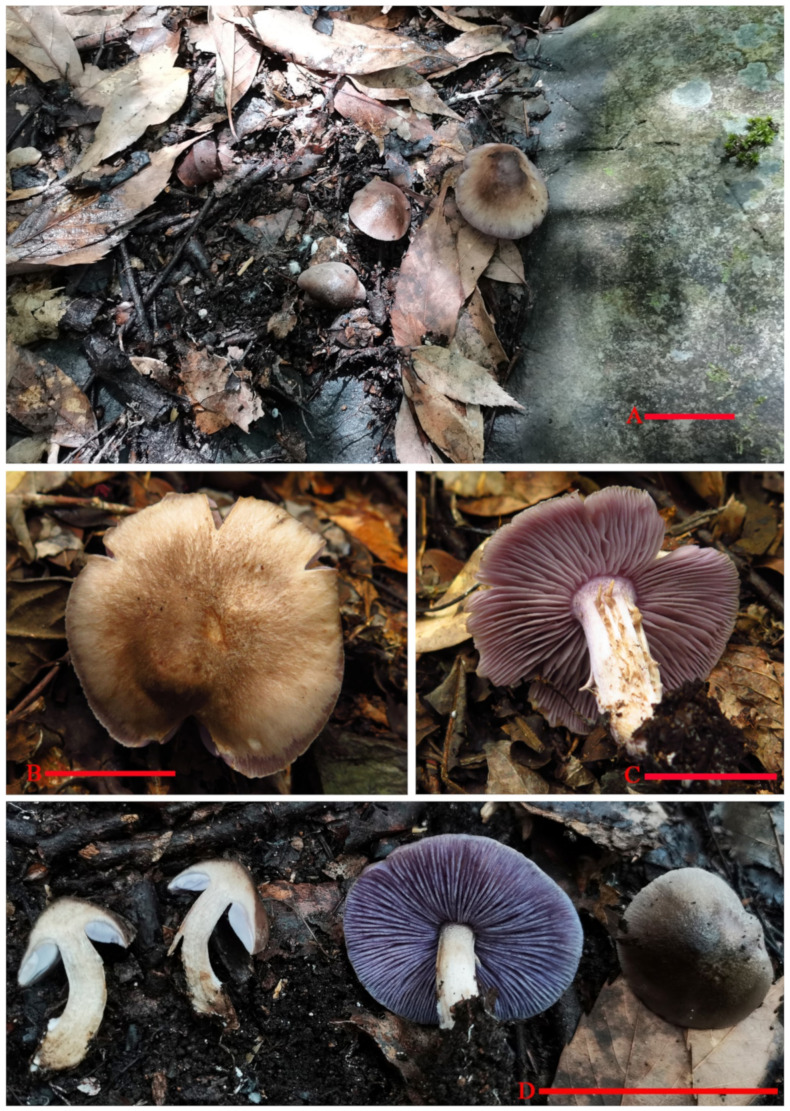
*Basidiomata* of *Asproinocybe sinensis*. Scale bar (**A**,**D**) = 5 cm; B, C = 2.5 cm. (**A**,**D**) from *HMJAU59025* (Holotype HMJAU); (**B**,**C**) from *M2020081289* (IBK!). Photos by Guang-fu Mou.

**Figure 4:**
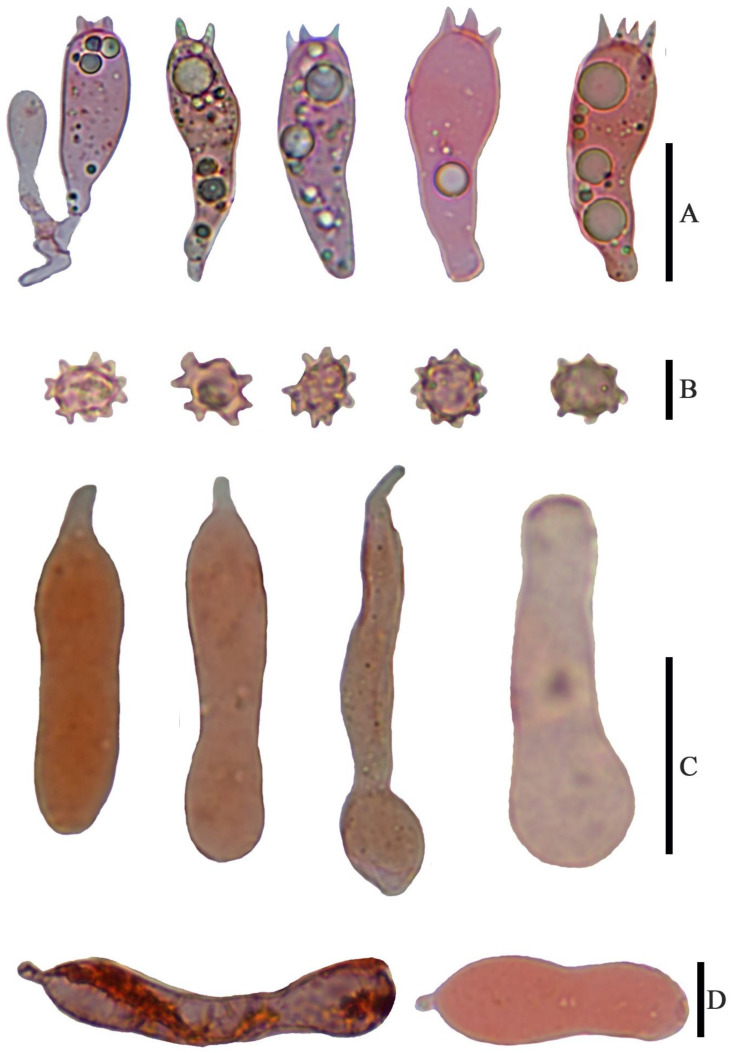
Microscopic features of *Asproinocybe sinensis,* from *HMJAU59025* (Holotype), stained with 1% Congo Red solution. (**A**) Basidia, *(***B**) Basidiospores, (**C**) Pleurocystidia, and (**D**) Cheilocystidia. Scale bar (**A**) =15 µm, (**B**) = 5 µm, (**C**) = 20 µm, and (**D**) = 10 µm. Photos by Guang-fu Mou.

**Figure 5:**
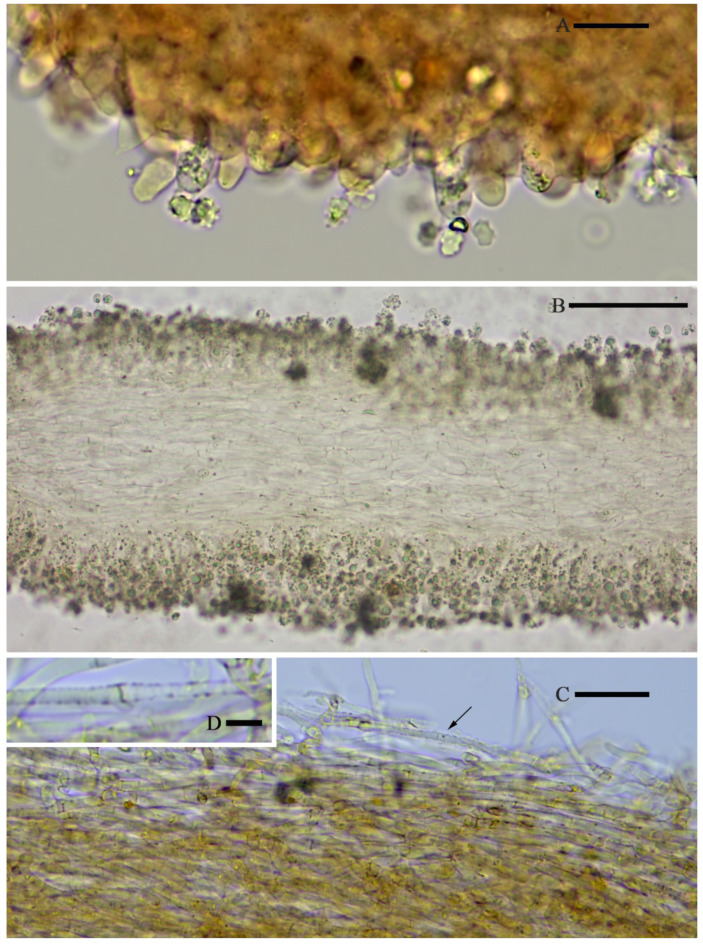
Microscopic features of *Asproinocybe sinensis,* from *HMJAU59025* (Holotype), in pure water. (**A**) Margin of lamella, (**B**) Hymenophoral trama, (**C**) Pileipellis, and (**D**) Hypha with incrustation, from Pileipellis. Scale bar (**A**) = 20 µm, (**B**) = 100 µm, (**C**) = 20 µm, and (**D**) = 5 µm. Photos by Guang-fu Mou.

**Figure 6:**
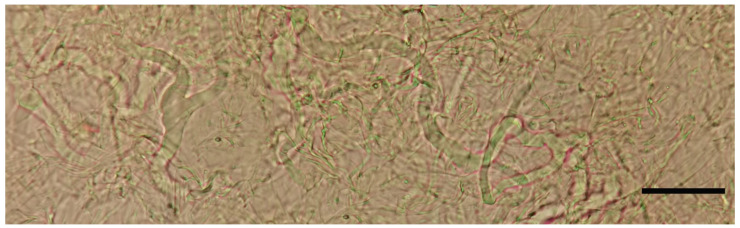
*Laticifers* of *Asproinocybe sinensis,* from *HMJAU59025* (Holotype), in pure water. Scale bar = 20 µm.

**Figure 7:**
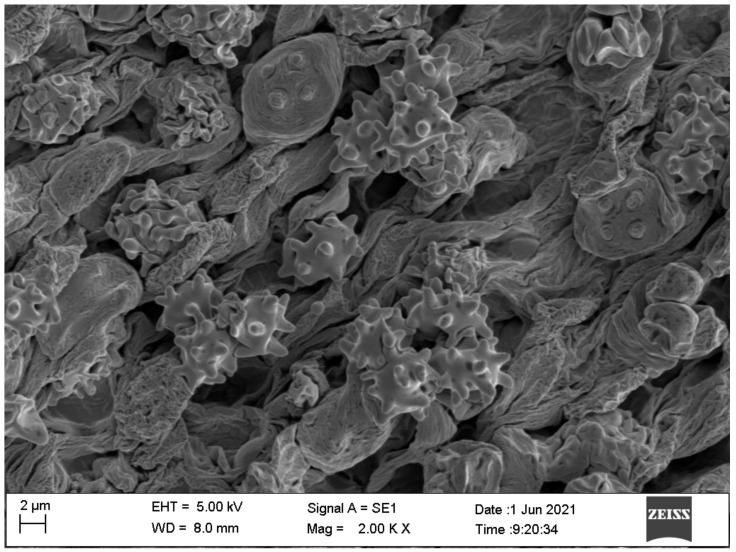
Basidiospores under SEM, from *Asproinocybe sinensis HMJAU59025* (Holotype).

***Asproinocybe sinensis*** T. Bau et G.F.Mou, **sp. nov.** (Figure 3, Figure 4, Figure 5, Figure 6 and Figure 7).

Mycobank No: MB841850.

Diagnosis: Differs from other known species of this genus by the central pileus being dark brown, with larger basidia (33 × 10 µm on average); *cheilocystidia* (30–40 × 8–10 µm) and *pleurocystidia* (38 × 9 µm on average) present and the apex, not branched; hyphae of *pileipellis* with fine incrustation.

Etymology: *sinensis* (Lat.): The locality of the type specimen was China.

Type: China, Guangxi province, Baise city, Leye country, Yachang Orchidaceae National Nature Reserve, 24°50′51.48″ N, 106°24′55.43″ E, elevation 1053 m, 12 August 2020, Guang-fu Mou HMJAU59025 (Holotype HMJAU!).

Description: *Basidiomata* tricholomatoid *habit*, solitary to gregarious (Figure 3). *Pileus* 35–55 mm in diam., broadly convex to subumbonate, becoming plane with age, margin smooth or with light short stripes, entire, incurved at first then straight, surface at first fibrillose-felted (due to very thin, white hairs), nonviscid; overall color from center to margin is dark brown (6E8), brown (6E6) to brownish orange (6C5), lilac grey (16C2) to violet (16C6). *Context* 3–8.5 mm thick, firm, whitish becoming greyish. *Lamellae* adnexed, close, 5 mm broad, with 1–2 tiers of lamellulae; margins smooth; lilac grey (16C2) to greyish violet (16C4) when immature, dull violet (16D4) to greyish violet (16D5) when mature, turning orange (6A7) to brownish orange (6C7) when damaged. *Stipe* to 43 mm long × 5–11 mm in diam., stout, central, equal, dry, violet white (16A2) to light violet (16A5), covered by white (16A1) to violet white (16A2) flocculose pruina, bruising dull. Base with white rhizomorphs. *Odor* not distinct. *Taste* not recorded. *Spore-print* white.

*Basidiospores* (6.5) 7.0–8.0 (9.0) × 4.8–6.0 (7.0) µm, 7.6 × 5.8 µm on average (Q = 1.1 − 1.5, Q_av_ = 1.3) [36/5/4], ornamentation not included), hyaline, colorless, inamyloid, thin-walled, densely tuberculate, ornamentation up to 1.0 µm high, sometimes with a single large oil-drop (Figure 4 and Figure 7). *Basidia* (25) 30–40 (44) × (8) 9–12 (13) µm, 33 × 10 µm on average [48/4/4], cylindric to narrowly clavate, thin-walled, colorless, usually with one to multiple oil drops, two or four sterigmate (Figure 4A). *Cheilocystidia* 30–40 × 8–10 µm, mostly ampullaceous, with a swollen base and a neck, acute or mucronate at apex, thin-walled, colorless (Figure 4D and Figure 5A). *Pleurocystidia* 29–44 (54) × 8–10 (13) µm, ampullaceous or clavate, with a swollen base and a neck, acute, mucronate or obtuse at apex, thin-walled, colorless (Figure 4C). *Hymenophoral trama* regular, hyphae thin-walled (Figure 5B). *Pileipellis* an undifferentiated cutis, hyphae 3–5 µm in diam., light yellow (4A4–4A5, under water), some hyphae with fine incrustation on surface (Figure 5C,D). *Laticifers* present, pale yellow (4A3), thick-walled, branched, 5–7.5 µm in diam. (Figure 6). *Clamp connections* present (Figure 5D).

Habitat: Scattered or gregarious in broad-leaved forest soil of karst areas, dominant tree species is *Cyclobalanopsis myrsinifolia*.

Known distribution: So far only known from Guangxi (China).

Additional material examined: China, Guangxi Province, Baise city, Leye country, Yachang Orchidaceae National Nature Reserve, 24°50′51.56″ N, 106°24′55.40″ E, elevation 1056 m, 12 August 2020, Guang-fu Mou HMJAU59026 (HMJAU!); China, Guangxi Province, Baise city, Leye country, Yachang Orchidaceae National Nature Reserve, 24°50′50.75″ N, 106°24′56.42″ E, elevation 1047 m, 12 August 2020, Guang-fu Mou M2020081289 (IBK!), China, Guangxi Province, Baise city, Leye country, Yachang Orchidaceae National Nature Reserve, 24°50′50.60″ N, 106°24′56.61″ E, elevation 1053 m, 12 August 2020, Guang-fu Mou M2020081292 (IBK!).

Notes: *Asproinocybe* is a small genus, characterized by the tricholomatoid *basidiomata* with distinctive purplish, violaceous, or lilac-vinaceous colors; spores with tuberculate ornamentation and present of the *laticifers*. Our specimens present these features. In 2020, Lebel et al. described two new species of this genus [6]. Our specimens are somewhat similar to *A. daleyae* in appearance: they all present a dark brown pileus. However, our specimens had larger *basidia* (33 × 10 µm vs. 20–30 × 5–7 µm), longer *cheilocystidia* (30–40 × 8–10 µm vs. 25–30 × 8–12 µm), and *pleurocystidia* (38 × 9 µm vs. 25–30 × 10–13 µm), and *hyphae* of the *pileipellis* had fine *incrustation*. The dark brown *pileus*, the presence of larger *cystidia*, and *hyphae* of *pileipellis* with fine *incrustation* can also be used to differentiate the rest of the known species. Our phylogenetic results (Figure 1 and Figure 2) agree with the morphological results.

***Tricholosporum Guzmán,*** Boln. Soc. mex. Micol. 9: 61 (1975).

Etymology: From cruciform basidiospores.

Type species: *Tricholosporum goniospermum* (Bres.) Guzmán ex T.J. Baroni.

Ecology and distribution: Scattered or gregarious on broad-leaved forest soil, widespread.

**Figure 8:**
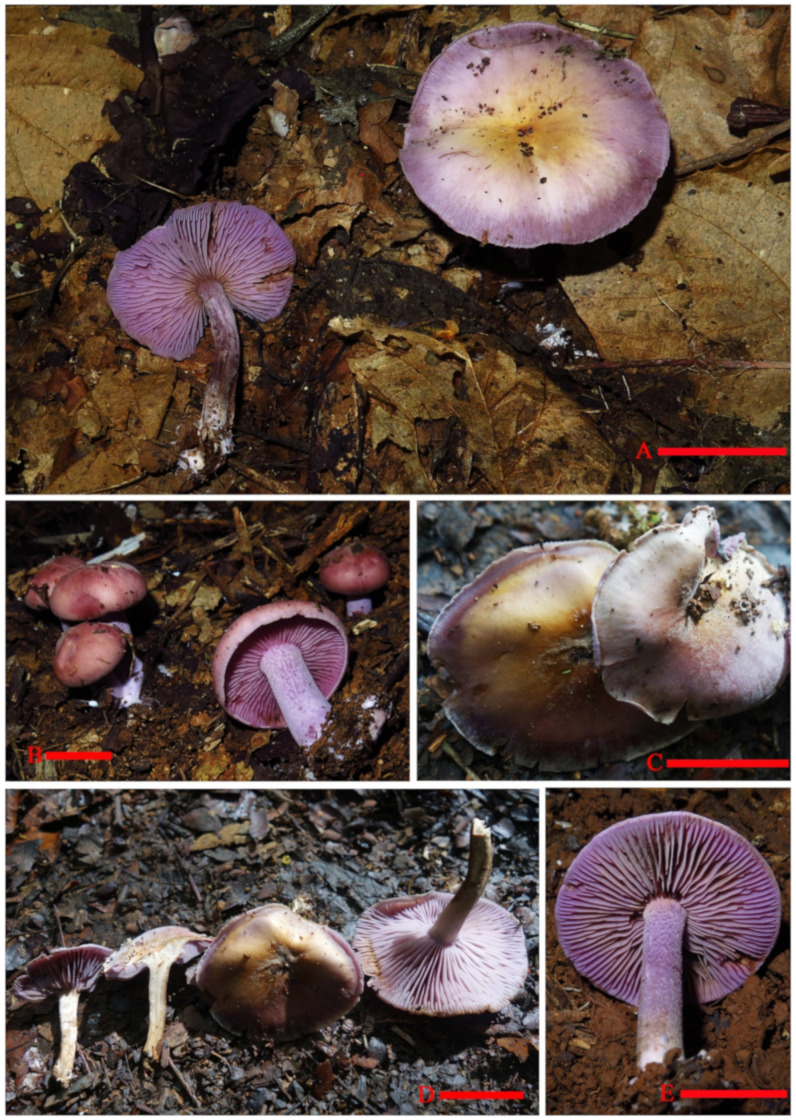
Basidiomata of *Tricholosporum guangxiense*. Scale bar (**A**–**D**) = 2.5 cm. A from *HMJAU59028* (Holotype HMJAU), (**B**) from *HMJAU59027*, (**C**,**D**) from *HMJAU59023*, and (**E**) from *M2021082208* (IBK). Photos by Guang-fu Mou.

**Figure 9:**
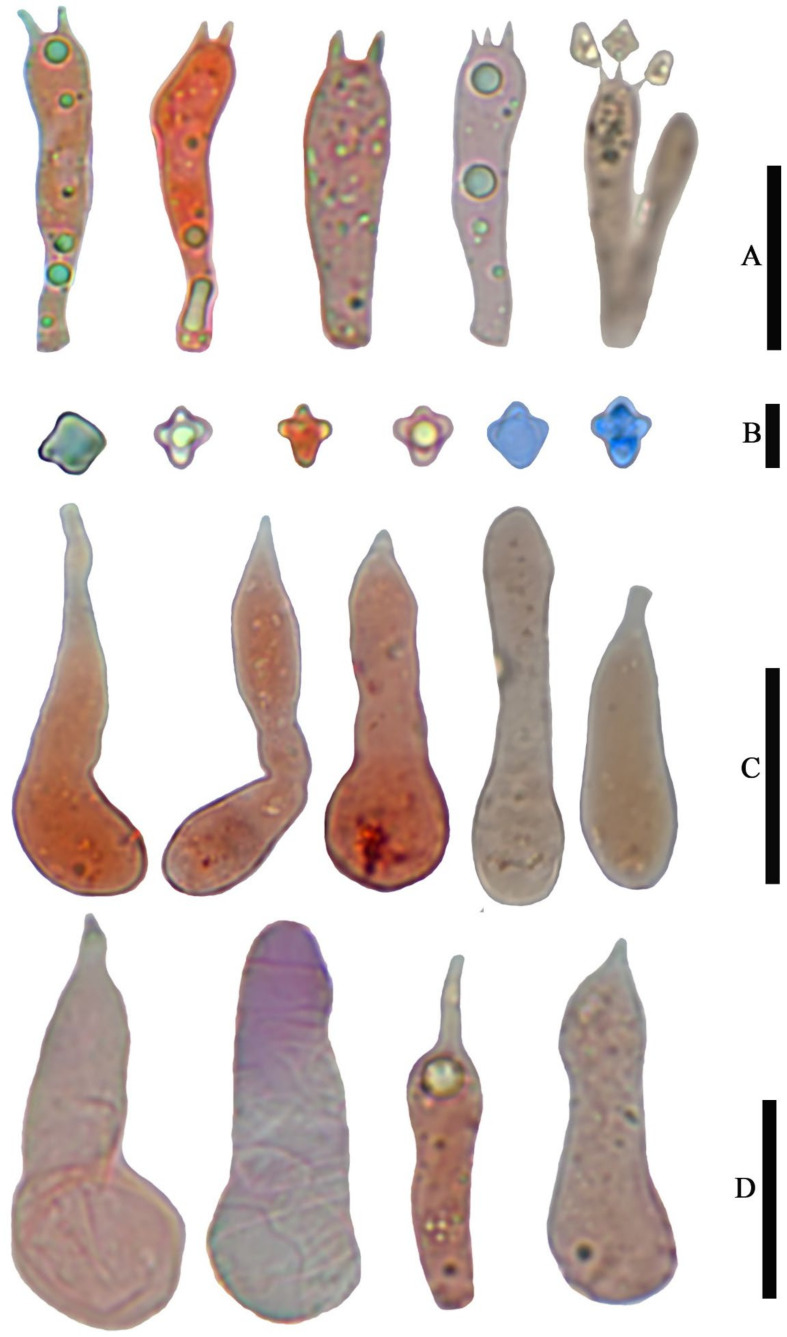
Microscopic features of *Tricholosporum guangxiense,* from *HMJAU59028* (Holotype). (**A**–**D**) stained with 1% Congo Red solution. (**B**) (from left to right) 1–2 in pure water, 3–4 stained with 1% Congo Red solution, 5–6 stained by Cotton blue. (**A**) Basidia, (**B**) Basidiospores, (**C**) Pleurocystidia, and (**D**) Cheilocystidia. Scale bar (**A**) = 15 µm, (**B**) = 5 µm, (**C**,**D**) = 20 µm. Photos by Guang-fu Mou.

**Figure 10:**
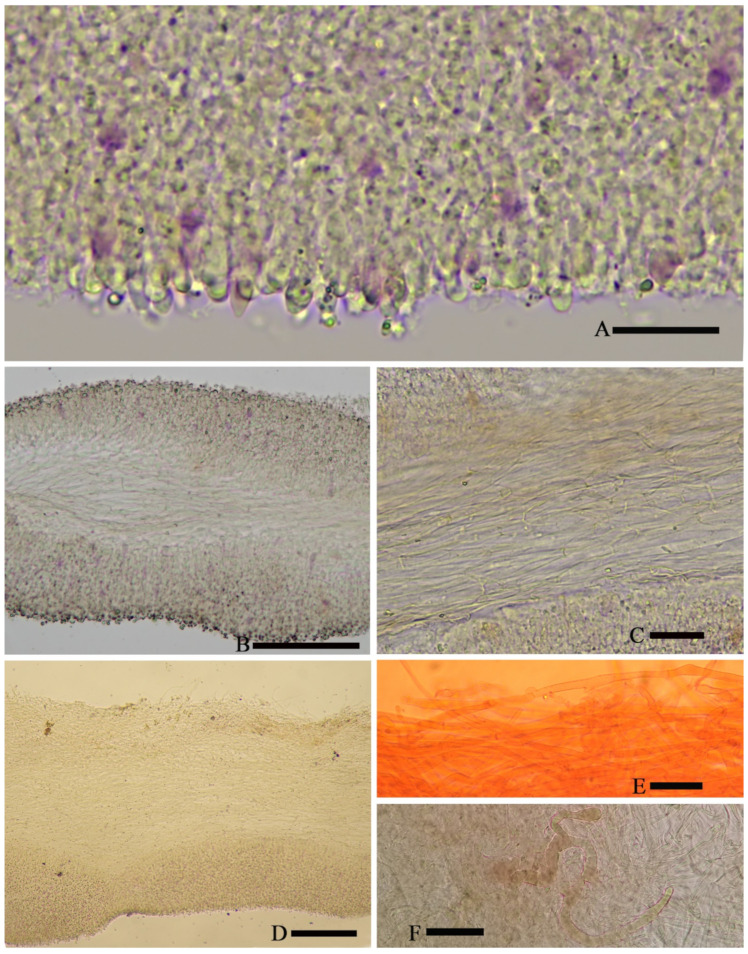
Microscopic features of *Tricholosporum guangxiense,* from *HMJAU59028* (Holotype). (**A**–**D**) F in pure water, (**E**) stained with 1% Congo Red solution. (**A**) Margin of lamella, (**B**,**C**) Hymenophoral trama, (**D**,**E**) Pileipellis and Hyphae of Pileipellis, and (**F**) Laticifer. Scale bar (**A**) = 15 µm, (**B**) = 5 µm, and (**C**–**F**) = 20 µm. Photos by Guang-fu Mou.

***Tricholosporum guangxiense*** T.Bau et G.F.Mou, **sp. nov.** (Figure 8, Figure 9 and Figure 10).

Mycobank No: MB841851.

Diagnosis: Differs from other known species of this genus by these combined features: *basidiomata* medium in size, *pileus* no color spots, central with obvious yellowish-brownish color when mature; *cheilocystidia* and *pleurocystidia* unfurcate and sometimes with purplish pigment; *spores* cyanophilous, not exceeding 7 µm, 5.4 × 4.7 µm on average.

Etymology: *guangxiense* (Lat.): The type specimen was obtained in Guangxi, China.

Type: China, Guangxi province, Chongzuo city, Ningming country, Nongang National Nature Reserve, 22°14′31.54″ N, 107°03′50.14″ E, elevation 167 m, 22 August 2021, *Guang-fu Mou HMJAU59028* (Holotype HMJAU!).

Description: *Basidiomata* tricholomatoid, solitary to gregarious (Figure 8). *Pileus* 43–55 mm in diam., convex to flat-hemispherical, becoming plane to depressed with age, margin smooth or with light short stripes, entire, incurved at first then straight to slightly reflexed, surface at first fibrillose-felted (due to very thin, white hairs), nonviscid; greyish ruby (12D4–12D5) when young, light lilac (16A5) to greyish violet (16C5) near margin and central becoming light orange (5A5) to brownish yellow (16C5) with age. *Context* up to 4 mm thick, firm, whitish. *Lamellae* emarginate, with small decurrent tooth, close, 5 mm in broad, with 2–3 tiers of lamellulae; margins smooth; light violet (1AC5) to violet (17A7), turning orange (6A7) to brownish orange (6B7) when damaged. *Stipe* 30 to 50 mm long × 5–8 mm in diam., stout, central, equal, dry, light violet (17A5) to violet (17A6), covered by violet white (16A2) to pale violet (16A3) pruina, bruising dull. *Basal* with white rhizomorphs. *Odor* not distinct. *Taste* not recorded. *Spore-print* white.

*Basidiospores* (4.0) 5.0–6.0 (7.0) × (3.6) 4.0–5.0 (5.4) µm, 5.4 × 4.7 µm on average (Q = 1.0–1.4, Q_av_ = 1.2) [38/5/5], cruciform, hyaline, colorless, inamyloid, cyanophilous (Figure 9B 5–6), thin-walled, usually with a single large oil drop (Figure 9B). *Basidia* (21) 23–28 (32) × 5–7 µm [48/3/3], cylindric to narrowly clavate, thin-walled, colorless, usually with one to multiple oil drops, two or four sterigmate (Figure 9A). *Cheilocystidia* (23) 27–36 (40) × 6–13 (14) µm, ampullaceous or clavate, with a swollen base and a neck, acute, mucronate or obtuse at apex, thin-walled, sometimes with purplish pink (14A5) to greyish magenta pigment (14D5) (Figure 9D and Figure 10A). *Pleurocystidia* (35) 40–50 (60) × (8) 9–13 (14) µm, ampullaceous or clavate, with a swollen base and a neck, acute, mucronate or obtuse at apex, sometimes curved, thin-walled, sometimes with grey-violet pigment (Figure 9C). *Hymenophoral trama* 148–243 µm broad, regular, hypha thin-walled (Figure 10B,C). *Pileipellis* an undifferentiated cutis, hyphae 4.6–5.8 µm in diam., colorless (Figure 10D,E). *Laticifers* present, pale yellow (4A3), thick-walled, branched, 5–10 µm in diam. (Figure 10F). *Clamp connections* present (Figure 10E).

Habitat: Scattered or gregarious on broad-leaved forest soil of karst areas; the associated tree species are *Streblus tonkinensis*, *Wendlandia uvariifolia*, *Sterculia monosperma*, *Musa balbisiana*, and *Heptapleurum* sp.

Known distribution: So far, only known in Guangxi (China).

Additional material examined: China, Guangxi Province, Chongzuo city, Ningming country, Nongang National Nature Reserve, 22°14′29.90″ N, 107°03′33.99″ E, elevation 263 m, 08 July 2018, *Guang-fu Mou HMJAU59023* (HMJAU!); China, Guangxi Province, Chongzuo city, Ningming country, Nongang National Nature Reserve, 22°14′31.63″ N, 107°03′50.59″ E, elevation 166 m, 22 August 2021, *Guang-fu Mou HMJAU59027* (HMJAU!). China, Guangxi Province, Chongzuo city, Ningming country, Nongang National Nature Reserve, 22°14′41.78″ N, 107°04′22.05″ E, elevation 145 m, 08 August 2021, *Guang-fu Mou M2021082219* (IBK!); China, Guangxi Province, Chongzuo city, Ningming country, Nongang National Nature Reserve, 22°14′32.55″ N, 107°03′55.02″ E, elevation 181 m, 8 August 2021, *Guang-fu Mou M2021082208* (IBK!).

Notes: Angelini et al., according to the size of the *basidiospores*, divided the species of *Tricholosporum* into two groups: group 1, the large-spored species with spores over 7 µm in length, and group 2, species with small spores, usually under 6 µm in length. According to the presence or absence of *hymenial cystidia* and whether grey-violet pigmentation was shown, they further divided group 2. Our specimens should obviously be categorized into group 2, subgroup 2.3: species with pigmented *hymenial cystidia*, which are grey-violet or brownish. Three species, *T. palmense, T. violaceum,* and *T. caraibicum,* were placed in this subgroup [4].

Our specimens differ from *T. violaceum* by their smaller and dry *pileus* (4.3–5.5 vs. 8–13 cm), thicker *context* (4 vs. 10–20 mm), narrower *lamellae* (5 vs. 15 mm), shorter *stipe* (5 vs. 5–11 cm), and larger *spores* (5.4 × 4.7 vs. 4.5 × 3.5 µm, on average) [4]; differ from *T. palmense* by the unfurcate *cystidia*; differ from *T. caraibicum* by the *pileus* lacking color spots, central with obvious yellowish-brownish color when mature, the larger *spores* (5.4 × 4.7 vs. 4.1 × 3.8 µm, on average), and being cyanophilous [4].

## 5. Discussion

The phylogenetic placement of the *Asproinocybe*/*Tricholosporum* clade has been discussed by Angelini et al. and Lebel et al. [6,20] but remains unresolved due to the poor sequencing of the species from this clade. Fortunately, we collected two new taxa from *Asproinocybe* and *Tricholosporum* and obtained another three specimens (one from the holotype) of the species *Tricholosporum haitangshanum*. Thus, we had 12 specimens for this study. Finally, we successfully extracted the DNA from 10 specimens, and a total of 43 sequences (15 from *Asproinocybe* and 28 from *Tricholosporum*) were obtained, including ITS, nrLSU, RPB1, RPB2, nrSSU, and TEF1-α sequences (see Table S1 in Supplementary Material, in bold). 

The overall topology in Figure 1 (the topology of the tree was obtained from Bayesian analysis) is consistent with the topologies published in previous studies [22,30,31,32,33,34,35,36,37,38], except for the positions of the genera *Bonomyces*, *Catathelasma,* and *Cleistocybe*. Sánchez-García et al., Alvarado et al. and Raj et al. also reported results similar to those of the present study [34,35,36,37]. Vizzini et al. explained that this difference in arrangement is due to the taxon sampling within *Catathelasma*, *Callistosporium*, and *Macrocybe* [38]. In the additional analyses, we obtained the same results as Vizzini et al. when increasing the sampling within *Catathelasma*, *Callistosporium*, and *Macrocybe* (not shown in the present study).

The relationships of the genera *Asproinocybe* and *Tricholosporum* have been discussed by many mycologists. Guzmán et al. and Baroni recognized *Tricholosporum* is distinct from *Asproinocybe* by the shape of spore; Lebel et al. believed that the relationship between *Tricholosporum* and *Asproinocybe* will remain problematic until further species of *Asproinocybe* are sequenced; Singer, Bohus, Alessio, Hongo, and Bon and Braiotta recognized recognized *Tricholosporum* is distinct from *Asproinocybe*, but considered *Tricholosporum* a synonym of *Tricholoma* in the Section Iorigida [2,3,4,6,11,12,13,14,15,16]. Morphologically, they have many common features—key features used to tell them apart are the spore shapes and the presence of *laticifers* [2]. *Laticifers* are rarely recorded in *Tricholosporum* and can even be considered as probably absent [2]. However, we truly observed both in *Tricholosporum guangxiense* (Figure 10F) and *Tricholosporum haitangshanum* (not shown in this study) but not so commonly as in *Asproinocybe.* Moreover, based on our results in Figure 1 and Figure 2, *Asproinocybe* and *Tricholosporum* do not form two independent clades. Should we consider *Tricholosporum* as a synonym of *Asproinocybe*? *Tricholosporum* cannot be a synonym of *Tricholoma* based on our results. However, based on our results in Figures S1 and S2, the species of *Asproinocybe* and *Tricholosporum* form two distinct clades, and based on the results in Figure 1, the species of *Asproinocybe* and *Tricholosporum* do not cross over.

Based on our results shown in Figure 1 and Figure 2, the species of *Asproinocybe lyophylloides*, *Asproinocybe daleyae*, or *Asproinocybe sinensis* form a monophyletic clade with the taxa of *Tricholosporum*. Should we treat them as independent genera? If so, no morphological delimitation is shown between the independent clades abovementioned. If we treat *Tricholosporum* as being distinct from *Asproinocybe*, it seems more reasonable. Thus, the *Tricholosporum* clade is a monophyletic clade with clear a morphological basis (from cruciform to stauriform spores). The taxa of *Asproinocybe* do not form a monophyletic clade in Figure 1 and Figure 2 but instead form a monophyletic clade in Figures S1 and S2 with a clear morphological basis (the tuberculate to stellate spores). A possible explanation for the *Asproinocybe* clades is that the present phylogenetic tree lacks of sampling between *Asproinocybe sinensis*, *A. daleyae*, and *A. lyophylloides*. Stronger evidence is needed to prove that *Tricholosporum* is a synonym of *Asproinocybe*; as such, we maintain the opinion that *Tricholosporum* is distinct from *Asproinocybe* due to the spore’s shape and the not-so-abundant *laticifers*.

We also noticed that *Tricholosporum* *haitangshanum* was close to *Tricholosporum goniospermum* in terms of both morphological and phylogenetic features. We will not analyze it until more specimens of *Tricholosporum goniospermum* have been studied.

At the family level, the clades of *Asproinocybe* and *Tricholosporum* were commonly placed Tricholomataceae s.l., Lyophyllaceae, and Entolomataceae [1,2,3,4,5,6,7,8,12,19,20]. Morphologically, those taxa have the tricholomatoid habit (especially in Tricholomataceae s.l. and Lyophyllaceae) and tuberculate spores. However, the species of *Asproinocybe* and *Tricholosporum* are always distinctive purplish, violaceous, or lilac-vinaceous colors, and the tuberculate spores are more remarkable. Some species in *Cortinarius* and *Inocybe* also have purplish basidiomata and tuberculate spores. However, their spores are brownish, and the results of Heaton and Kropp refute the possible relationships [19]. Another possible group is the Clitocybeae, which includes the genus *Lepista,* which could be similar to the species of *Asproinocybe* and *Tricholosporum.* Our phylogenetic analysis included these species: they were clearly separated and could be easily discriminated under a microscope. Another important feature indicating *Asproinocybe* and *Tricholosporum* as a new family is that they have *laticifers*.

Morphologically, the species of *Asproinocybe* and *Tricholosporum* are somewhat similar to those of Callistosporiaceae. They have the same features: tricholomatoid *habit*, *veils* absent; *lamellae* adnate, adnexed, sinuate, emarginated to decurrent; *spore print* white, *spores* cyanophilous or acyanophilous, thin-walled; *hymenophoral trama* regular; and *pileipellis* arranged as a cutis [38]. All the species of *Asproinocybe* and *Tricholosporum* are more or less purplish, violaceous, or lilac-vinaceous; the species of Callistosporiaceae can also have similar coloration, such as *Callistosporium elegans*.

The species of Callistosporiaceae grow in soil or rotten wood and aresaprotrophic or ectomycorrhizal [38]. The species of *Asproinocybe* and *Tricholosporum* also grow in soil. We do not know if they form mycorrhizal relationships with plants, but they usually have white rhizomorphs. Recently, *Asproinocybe lactifera* was reported as an ectomycorrhizae fungus [46]. This is worthy of further study, but finding species of Asproinocybaceae is challenging.

Compared to Callistosporiaceae, *Asproinocybe* and *Tricholosporum* have some unique features, such as the basidiomata being distinctively purplish, violaceous, or lilac-vinaceous, spores tuberculate to stellate (*Asproinocybe*) or cruciform to stauriform (*Tricholosporum*), *laticifers* present, and the *lamellae* bruising reddish or pale brown when damaged [1,2,3,4,5,6,7,8].

Asproinocybaceae was an important lineage in the evolution of agarics. The presence of *laticifers*, *lamellae* bruising reddish, and *spores* with ornamentation and ectomycorrhizae [46] led to us link it with Russulaceae, *Lactarius*. The species of *Lactarius* also have *basidiomata* shapes similar to those of species of Asproinocybaceae, but the spores of *Lactarius* are amyloid. The relationship of the spore shapes between *Tricholosporum* and Entolomataceae was discussed by Angelini et al. [20]. According to the results reported by David et al. [31], the spore walls forming the ornamentation of Entolomataceae may not be homologous to those of other tricholomatoid species with bumped spores [20]. Our study confirms that *Tricholosporum* is included in a new clade that is different from the tricholomatoid species previously known. Thus, we may have to reconsider the homology of spores between Asproinocybaceae and Entolomataceae.

Species from Callistosporiaceae are saprotrophic or ectomycorrhizal [38]; as the sister family, species from Asproinocybaceae may be ectomycorrhizal [46]. The new species proposed here were collected from karst areas, where the soil is thin and infertile, where stony desertification is common, and where it is difficult for the vegetation to recover. If the species from Asproinocybaceae are ectomycorrhizal, they could help with vegetation recovery in areas suffering from stony desertification using mycorrhizal techniques. Species from Callistosporiaceae and Asproinocybaceae may provide suitable study material for explaining the evolution of mycorrhizal and nonmycorrhizal fungi.

## Data Availability

Publicly available datasets were analyzed in this study. This data can be found here: https://www.ncbi.nlm.nih.gov (accessed on 1 October 2021); https://www.mycobank.org (accessed on 28 October 2021).

## Supplementary Materials

The following are available online at https://www.mdpi.com/article/10.3390/jof7121086/s1. Table S1: Specimens used in phylogenetic analysis and GenBank codes. Newly sequenced collections are in bold. Figure S1: Phylogenetic tree inferred from partial ITS + LSU sequences showing phylogenetic relationships of *Asproinocybe* and *Tricholosporum*. Bayesian inference (BPP ≥ 0.90) and maximum likelihood support values (ML ≥ 70) are shown (BPP/ML). Figure S2: Phylogenetic tree inferred from partial ITS + LSU sequences showing phylogenetic relationships of *Asproinocybe* and *Tricholosporum*. Bayesian inference (BPP ≥ 0.90) and maximum likelihood support values (ML ≥ 70) are shown (BPP/ML).
