# Supplementary material for: Asproinocybaceae fam. nov. (Agaricales, Agaricomycetes) for Accommodating the Genera Asproinocybe and Tricholosporum, and Description of Asproinocybe sinensis and Tricholosporum guangxiense sp. nov
Source: J Fungi (Basel). 2021 Dec 17;7(12):1086. doi: 10.3390/jof7121086 (PMC8707192; doi:10.3390/jof7121086)
Supplement: Supplementary file 1 [file jof-07-01086-s001.zip › 11.23-supplmentary.pdf]

Table S1. Specimens used in phylogenetic analysis and GenBank codes. Newly sequenced collections are in bold.

Species	Voucher/ Strain	GenBank Accession no.						
		ITS	nrLSU	RPB2	TEF1-α	RPB1	nrSSU	Reference
Asproinocybe sinensis	**HMJAU59025 Holotype**	**OK377049**[*#]	**OK377052**[*#]	**OK625400**	**OK625330**	**OK625397**	**OK377041**	**Present study**
Asproinocybe sinensis	**HMJAU59026**	**OK377048**[*#]	**OK377051**[*#]	**OK625401**	**OK625331**	**OK625398**	**OK377040**	**Present study**
Asproinocybe sinensis	**M2020081289**	-	**OK576386**[*#]	-	**OK625337**	-	**OK624826**	**Present study**
Asproinocybe daleyae	PDD 106796	MN275025[*&]	MN275033[*&]	-	-	-	-	[6]
Asproinocybe lyophylloides	PERTH 4163559	MN275018[*&]	MN275027[*&]	-	-	-	-	[6]
Asproinocybe lyophylloides	MEL2432747	MN275015[*&]	MN275028[*&]	-	-	-	-	[6]
Asproinocybe lyophylloides	PERTH 8477001	MN275022[*&]	MN275030[*&]	-	-	-	-	[6]
Asproinocybe lyophylloides	PERTH E6292	MN275023[*&]	MN275031[*&]	-	-	-	-	[6]
Ampulloclitocybe clavipes	AFTOL-ID 542	AY789080	AY639881	AY780937	AY881022	AY788848	-	[35,36]
Anupama indica	AMH10033	MH989590	MH989586	MH992117	-	-	-	[37,38]
Anupama indica	CAL1725	MH989587	MH989583	-	-	-	-	[37,38]
Asterophora lycoperdoides	CBS170.86	AF357037	-	DQ367431	DQ367424	EF421021	AF357109	[32]
Bonomyces afrosinopicus	LIP:LYK13040015	MG696613	MG696624	MG702593	MG702590	-	MG696621	[36]
Callistosporium graminicolor	AFTOL978	DQ484065	AY745702	-	-	-	-	[35,36]
Callistosporium imbricatum	TJB9847	HM105568	HM105568	HM105567	-	-	-	[35,38]
Callistosporium imbricatum	SFSU:DED 8232	MF100955	MN017456	-	-	-	MN017570	[38]
Callistosporium luteoolivaceum	18231 (AMB)	MN017518[*#&]	MN017459[*#&]	-	-	-	-	[38]
Callistosporium praemultifolium	SFSU:DED 8238	MF100956	MN017464	MN018844	-	-	MN017575	[38]
Callistosporium xanthophyllum	IB19770276	AF325667[*#&]	AF261406[*#&]	-	-	-	-	[37]
Catathelasma imperiale	CORT:11CA01A	-	KR869941	KC816994	KC816900	-	KR869913	NCBI
Catathelasma imperiale	HKAS 84315	MK909096	MK909113	-	MK909083	-	-	NCBI
Cleistocybe carneogrisea	TENN:063842	-	HQ728527	-	-	-	HQ728528	[38]
Cleistocybe vernalis	ADP 050506 (WTU)	EF416917	EF416916	-	-	-	-	[38]
Clitocybe candicans	AFTOL-ID 541	DQ202268	AY645055	DQ385881	DQ408149	DQ447891	-	[32]
Clitocybe dealbata	IE-BSG-HC95.cp3	AF357061	AF223175	DQ825407	EF421080	DQ825414	AF357138	[32,34]
Clitocybe eccentrica	G0084/ PBM3851	MG663292	MK277727	-	-	-	-	NCBI

Table S1. Cont.

Species	Voucher/ Strain	GenBank Accession no.						
		ITS	nrLSU	RPB2	TEF1-α	RPB1	nrSSU	Reference
Clitocybe subditopoda	AFTOL-ID 533	DQ202269	AY691889	AY780942	DQ408150	DQ447892	-	[32]
Clitopilus brunneiceps	KUN-HKAS 80211	MN061293	MN065682	MN148121	MN166232	-	-	[22]
Clitopilus brunneiceps	KUN-HKAS 104510	MN061295	MN065684	MN148123	MN166234	-	-	[22]
Clitopilus rugosiceps	KUN-HKAS73232	MN061305	MN065695	MN148132	MN166244	-	-	[22]
Clitopilus rugosiceps	KUN-HKAS 107044	MT345046	MT345051	-	-	-	-	[22]
Clitopilus yunnanensis	KUN-HKAS104518	MN061308	MN065698	MN148136	MN166247	-	-	[22]
Clitopilus yunnanensis	HMJAU 24677	MN061309	MN065699	MN148116	MN166248	-	-	[22]
Clitopilus sinoapalus	KUN-HKAS101191	MN061322	MN065711	MN148151	MN166261	-	-	[22]
Clitopilus sinoapalus	KUN-HKAS 82230	MN061320	MN065712	MN148148	-	-	-	[22]
Clitopilus umbilicatus	KUN-HKAS80310	MN061324	MN065716	MN148153	MN166263	-	-	[22]
Clitopilus umbilicatus	KUN-HKAS 80945	MN061326	MN065718	MN148155	MN166265	-	-	[22]
Clitopilus prunulus	VHAs07/02	EF421107	EF421092	DQ825408	EF421086	DQ825416	EF421097	[32]
Clitocella orientalis	KUN-HKAS78763	-	MN065728	MN148165	MN166276	-	-	[22]
Clitocella orientalis	KUN-HKAS 77899	-	MN065725	MN148162	MN166273	-	-	[22]
Clitocella mundula	O:O-F71544	-	-	KC816950	KC816860	-	-	[22]
Clitocella mundula	HMJAU 7275	MN061331	MN065723	MN148160	MN166271	-	-	[22]
Clitocella fallax	O:O-F88953	-	-	KC816936	KC816845	-	-	[22]
Clitocella fallax	K: K(M) 116541	-	-	KC816938	KC816847	-	-	[22]
Clitocella fallax	CBS 605.79	AF357017	AF223165	-	-	-	AF357084	NCBI
Clitopilopsis albida	KUN-HKAS104519	MN061335	MN065730	MN148167	MN166278	-	-	[22]
Clitopilopsis albida	KUN-HKAS104520	MN061336	MN065731	MN148168	MN166279	-	-	[22]
Collybia tuberosa	DUKE-AOM191061	AF274376	AF261385	KP255481	KP255474	KP255479	-	[32]
Dendrocollybia racemosa	DUKE-DEB5575	DQ825425	AF042598	DQ825409	KP255476	DQ825417	KP255472	[32]
Entocybe nitida	24	KC710122	GQ289175	-	-	-	-	[6]
Entocybe trachyospora	414	KC710121	GQ289199	-	-	-	GQ289339	[31]
Entoloma turbidum	PRM915266	FJ824815	GQ244337	-	-	-	-	[6]
Entoloma turbidum	27	KC710060	GQ289201	GQ289269	-	-	GQ289341	[31]
Entoloma hainanense	GDGM 27990	NR_137783	NG_059212	-	-	-	-	NCBI

Table S1. Cont.								
**Species**	**Voucher/ Strain**	**GenBank Accession no.**						
		**ITS**	**nrLSU**	**RPB2**	**TEF1-α**	**RPB1**	**nrSSU**	**Reference**
Entoloma pallidocarpum	GDGM 28828	JQ320106	JQ410331	JQ993080	-	-	JQ993074	NCBI
Entoloma perbloxamii	71	KC710117	GQ289178	GQ289249	-	-	GQ289318	[31]
Entoloma porphyrophaeum	VHAs09/02	EF421111	-	-	EF421090	EF421053	EF421101	[32]
Entoloma porphyrophaeum	TB6957	-	AF261290	EF421020	-	-	-	[32]
Entoloma sericeonitidum	TB7144	EF421108	AF261315	EF421016	EF421087	EF421049	EF421098	[32]
Entoloma sericeum	VHAs03/02	DQ367430	DQ367423	DQ367435	DQ367428	DQ825424	EF421099	[32]
Entoloma strictius	DUKE-JM96/10	EF421109	AF042620	EF421017	EF421088	EF421050	EF421100	[32]
Gerhardtia sp.	HC01/025	EF421103	EF421091	EF420994	EF421060	EF421028	EF421093	[32]
Guyanagarika pakaraimensis	MCA4776	KX092085	KX092104	KX092139	-	-	-	[34,37]
Guyanagarika anomala	TH7419	KX092096	KX092110	KX092147	-	-	-	[37]
Infundibulicybe geotropa	ALV4344	KT122792	KT122793	-	-	-	-	[35,38]
Infundibulicybe gibba	AFTOL-ID 1508	DQ490635	DQ457682	DQ472727	-	DQ447913	-	[35]
Lepista irina	AFTOL-ID 815	DQ221109*	DQ234538*	DQ385885	DQ028591	DQ447919	-	[35]
Lepista nebularis	CBS362.65	AF357063	AF223217	EF421011	EF421081	DQ825415	AF357142	[35]
Lepista nuda	DUKE-RV84/1	AF357062*	AF042624*	EF421012	EF421082	EF421045	-	[32]
Leucopaxillus subzonalis	GB:0087013	KP453695	KJ417208	KJ424385	-	-	-	[33]
Lyophyllum connatum	DUKE-JM 90c	EF421104	AF042590	EF420995	EF421061	EF421029	_	[32]
Lyophyllum decastes	JM87/16	AF357059	AF042583	DQ367433	DQ367426	-	AF357136	[32]
Lyophyllum decastes	JM-Hon/k (T4)	AF357060	AF357078	EF421001	EF421067	F421035	-	[32]
Lyophyllum favrei	IE-BSG-HC96cp4	EF421102	AF223184	EF420990	EF421056	EF421024	AF357104	[32]
Lyophyllum leucophaeatum	IE-BSG-HAe251.97	AF357032	AF223202	DQ367434	DQ367427	DQ825419	AF357101	[32]
Lyophyllum semitale	IE-BSG-HC85/13	AF357049	AF042581	EF421002	EF421068	EF421036	AF357125	[32]
Macrocybe sardoa	29083a (MCVE)	MN017542	MN017481	-	-	-	MN017588	[38]
Macrocybe titans	58974 (FLAS-F)	MN017545	MN017484	-	MN026908	-	MN017589	[38]
Musumecia bettlachensis	TO HG2284	JF926520	JF926521	KJ681060	KJ681082	-	KJ681069	[35]
Musumecia vermicularis	LUG18975	-	KJ681037	KJ681061	KJ681083	-	KJ681070	[35]
Neohygrophorus angelesianus	AFTOL-ID 1719	DQ494678	DQ470814	-	-	-	-	[30,35]
Ossicaulis lignatilis	DUKE-D604(VT)	DQ825426	AF261397	DQ825410	EF421072	DQ825420	EF421094	[32]

Table S1. Cont.

Species	Voucher/ Strain	GenBank Accession no.						
		ITS	nrLSU	RPB2	TEF1-α	RPB1	nrSSU	Reference
Pogonoloma macrocephalum	TENN:037026	KP453700	KJ417209	-	-	-	KJ417168	[35]
Pogonoloma spinulosum	K(M):107286	KP453705	KJ417238	KJ424401	-	-	KU058571	[35]
Porpoloma terreum	CONC: F0030	KJ417306	KJ417216	-	-	-	-	[33]
Porpoloma terreum	REH5830	KJ417305	KJ417215	KJ424391	-	-	-	[33]
Porpoloma portentosum	MES531	KJ417298	KJ417210	KJ424386	-	-	-	[33]
Porpoloma sp.	DUKE-PR3995	EF421106	AF261395	EF421013	EF421083	EF421046	EF421095	[32]
Pseudoclitocybe cyathiformis	AFTOL 1998	-	EF551313	GU187815	GU187742	-	GU187659	[35]
Pseudoclitopilus rhodoleucus	GB:0110967	KP453696	KJ417218	KJ424393	-	-	KU058577	[35]
Pseudolaccaria fellea	006240 (WTU)	MN017549	MN017487	-	MN026911	-	MN017591	[38]
Pseudolaccaria pachyphylla	TR gmb 00672	NR_153455	NG_060151	-	-	-	-	[38]
Pseudoarmillariella ectypoides	AFTOL-ID 1557	DQ192175	DQ154111	DQ474127	-	DQ516076	-	[35]
Pseudoomphalina kalchbrenneri	T0 HG 22102011C	KR818913	KR818915	-	-	-	-	[35]
Rhodocybe truncata	CBS482/50	EF421110	AF223167	EF421019	KP255478	EF421052	AF357086	[32]
Suillus pictus	AFTOL 717	AY854069	AY684154	AY786066	AY883429	AY858965	AY662659	[35]
Tephrocybe boudieri	IE-BSG-BSI96/84	AF357047	AF223204	DQ825411	EF421070	DQ825421	AF357122	[32]
Tephrocybe striaepilea	FR2014091	KP192644	-	KP192524	-	-	-	NCBI
Termitomyces microcarpus	DUKE-PRU3900	AF357023	AF042587	EF421009	EF421077	EF421043	AF357092	[32]
Tricholoma myomyces	DUKE- KMS589	DQ825428	U76459	DQ367436	DQ367429	DQ842013	EF421096	[32]
Tricholoma terreum	C59300	EU653300	EU653304	-	-	-	JN389400	NCBI
Tricholomella constricta	IE-BSG-HC84/75	AF357036	AF223188	DQ825412	EF421079	DQ825422	AF357105	[32]
Tricholosporum goniospermum	AR122	KU559861[*#&]	-	KU559863	-	-	-	[20]
Tricholosporum goniospermum	MS41	KU559844[*#&]	-	-	-	-	-	[20]
Tricholosporum goniospermum	PeruMyc2084	MT707943[*#&]	-	-	-	-	-	NCBI
Tricholosporum porphyrophyllum	HMJAU24949	KU954553[* #&]	KU954556[*#&]	KX397356	-	-	-	NCBI
Tricholosporum porphyrophyllum	H6849	KU954554[*#&]	KU954558[*#&]	KX397357	-	-	-	NCBI
Tricholosporum porphyrophyllum	KUBOT-KRMK-2020-94	MW485792[*#&]	MW485793[*#&]	-	-	-	-	NCBI
Tricholosporum guangxiense	**HMJAU59023**	**OK377045**[*#&]	**OK377053**[*#&]	**OK625399**	**OK625329**	-	**OK624824**	**Present study**
Tricholosporum guangxiense	**HMJAU59027**	**OK377046**[*#&]	**OK377055**[*#&]	**OK625402**	**OK625332**	-	**OK377042**	**Present study**

Table S1. Cont.								
**Species**	**Voucher/ Strain**	**GenBank Accession no.**						
		**ITS**	**nrLSU**	**RPB2**	**TEF1-α**	**RPB1**	**nrSSU**	**Reference**
Tricholosporum guangxiense	**HMJAU59028 Holotype**	**OK377047*#&**	**OK377056*#&**	**OK625403**	**OK625333**	-	**OK377043**	**Present study**
Tricholosporum guangxiense	**M2021082219 (IBK)**	-	**OK576387*#&**	-	**OK625335**	-	**OK624827**	**Present study**
Tricholosporum haitangshanum	**HMJAU59029**	**OK377050*#&**	**OK576384*#&**	**-**	**OK625334**	-	**-**	**Present study**
Tricholosporum haitangshanum	**HMJAU33972 Holotype**	**OK576388*#&**	**OK576383*#&**	**-**	**OK625338**	**-**	**OK624823**	**Present study**
Tricholosporum haitangshanum	**XJZ20160817**	**-**	**OK576385*#&**	**-**	**OK625336**	**-**	**OK624825**	**Present study**
Tricholosporum sp.	LG218-1	MF538719*#&	-	-	-	-	-	NCBI
Tricholosporum sp.	LG218-9	MF538721*#&	-	-	-	-	-	NCBI
Xerophorus olivascens	18224 (AMB)	MN017556	MN017494	-	-	-	-	[38]
Xerophorus olivascens	18226 (AMB)	MN017558	MN017496	MN018856	MN026916	-	-	[38]

Note:

Sequences (GenBank Accession no.) marked with *, were used in the phylogenetic analyses of **Figure 2.**

Sequences (GenBank Accession no.) marked with #, were used in the phylogenetic analyses of **Figure S1.**

Sequences (GenBank Accession no.) marked with &, were used in the phylogenetic analyses of **Figure S2.**

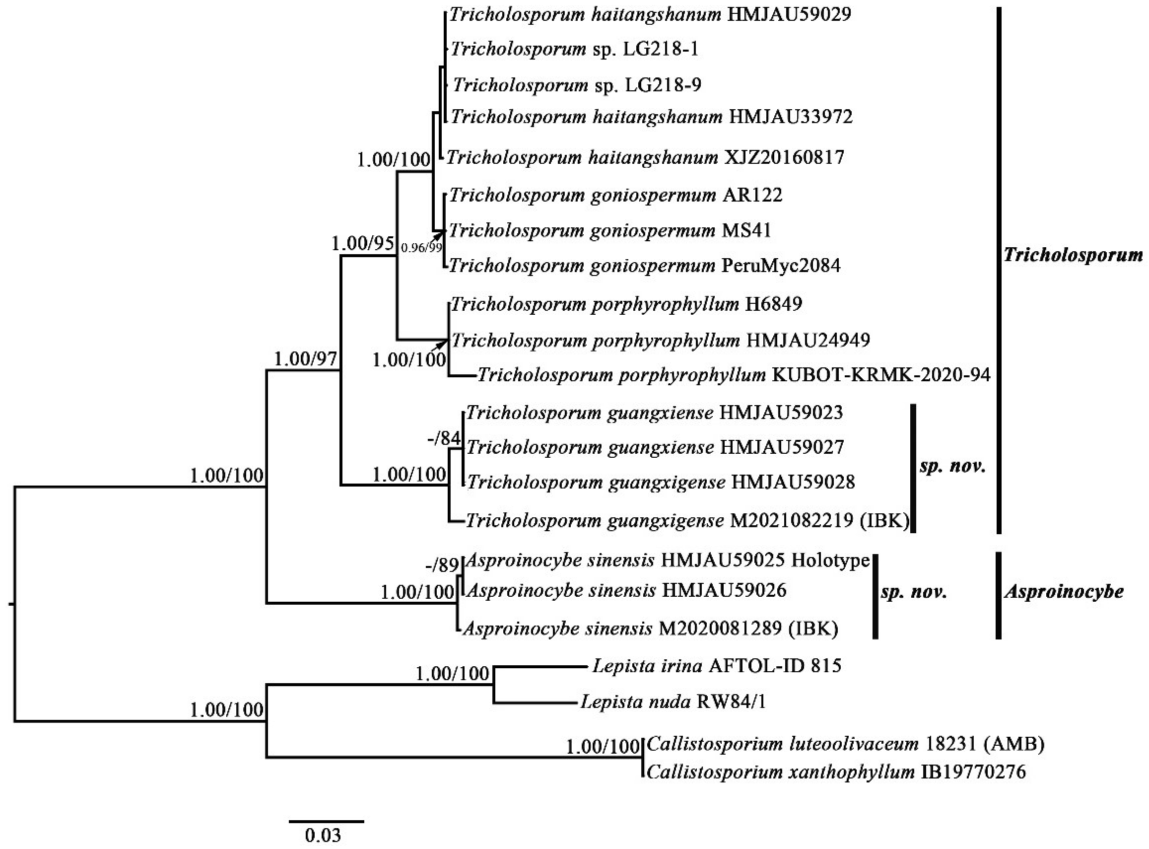

Figure S1. Phylogenetic tree inferred from partial ITS+LSU sequences showing phylogenetic relationships of *Asproinocybe* and *Tricholosporum*. Bayesian inference (BPP≥0.90) and maximum likelihood support values (ML≥70) are shown (BPP/ML).

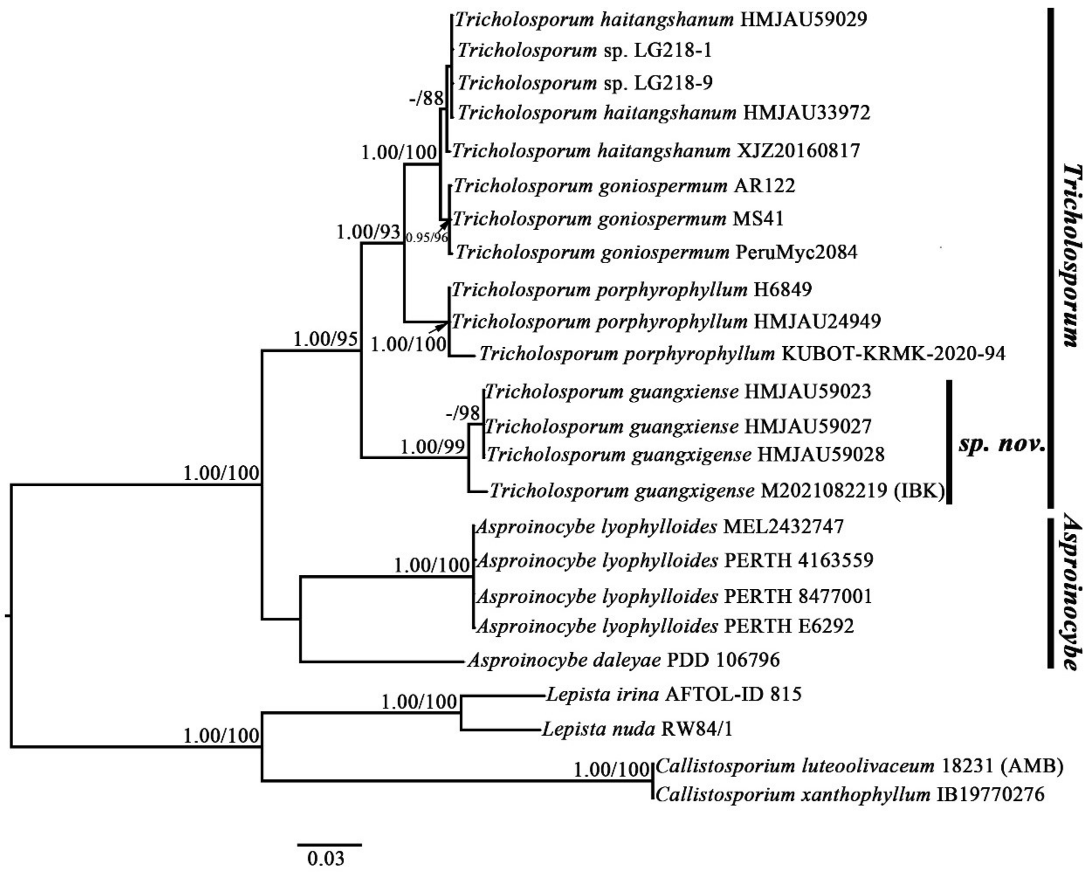

Figure S2. Phylogenetic tree inferred from partial ITS+LSU sequences showing phylogenetic relationships of *Asproinocybe* and *Tricholosporum*. Bayesian inference (BPP≥0.90) and maximum likelihood support values (ML≥70) are shown (BPP/ML)
